# Regional Brain Correlates of Beta Bursts in Health and Psychosis: A Concurrent Electroencephalography and Functional Magnetic Resonance Imaging Study

**DOI:** 10.1016/j.bpsc.2020.10.018

**Published:** 2021-12

**Authors:** Paul M. Briley, Elizabeth B. Liddle, Molly Simmonite, Marije Jansen, Thomas P. White, Vijender Balain, Lena Palaniyappan, Richard Bowtell, Karen J. Mullinger, Peter F. Liddle

**Affiliations:** a Institute of Mental Health, Division of Psychiatry and Applied Psychology, School of Medicine, University of Nottingham, University Park, Nottingham, United Kingdom; b Sir Peter Mansfield Imaging Centre, University of Nottingham, University Park, Nottingham, United Kingdom; c Nottinghamshire Healthcare NHS Foundation Trust, Mapperley, Nottingham, United Kingdom; d School of Psychology, University of Birmingham, Edgbaston, Birmingham, United Kingdom; e Burnaby Centre for Mental Health and Addictions, Burnaby, British Columbia, Canada; f Department of Psychiatry, University of Western Ontario, London, Ontario, Canada; g Robarts Research Institute, University of Western Ontario, London, Ontario, Canada; h Lawson Health Research Institute, London, Ontario, Canada

**Keywords:** Concurrent EEG/fMRI, Persisting psychotic illness, Post-movement beta rebound, Psychosis, Transient beta oscillations, Working memory

## Abstract

**Background:**

There is emerging evidence for abnormal beta oscillations in psychosis. Beta oscillations are likely to play a key role in the coordination of sensorimotor information that is crucial to healthy mental function. Growing evidence suggests that beta oscillations typically manifest as transient beta bursts that increase in probability following a motor response, observable as post-movement beta rebound. Evidence indicates that post-movement beta rebound is attenuated in psychosis, with greater attenuation associated with greater symptom severity and impairment. Delineating the functional role of beta bursts therefore may be key to understanding the mechanisms underlying persistent psychotic illness.

**Methods:**

We used concurrent electroencephalography and functional magnetic resonance imaging to identify blood oxygen level–dependent correlates of beta bursts during the n-back working memory task and intervening rest periods in healthy control participants (*n* = 30) and patients with psychosis (*n* = 48).

**Results:**

During both task blocks and intervening rest periods, beta bursts phasically activated regions implicated in task-relevant content while suppressing currently tonically active regions. Patients showed attenuated post-movement beta rebound that was associated with persisting disorganization symptoms as well as impairments in cognition and role function. Patients also showed greater task-related reductions in overall beta burst rate and showed greater, more extensive, beta burst–related blood oxygen level–dependent activation.

**Conclusions:**

Our evidence supports a model in which beta bursts reactivate latently maintained sensorimotor information and are dysregulated and inefficient in psychosis. We propose that abnormalities in the mechanisms by which beta bursts coordinate reactivation of contextually appropriate content can manifest as disorganization, working memory deficits, and inaccurate forward models and may underlie a core deficit associated with persisting symptoms and impairment.

SEE COMMENTARY ON PAGE 1121

Emerging evidence indicates that beta oscillations are abnormal in a range of neuropsychiatric conditions, including schizophrenia (1, 2, 3, 4, 5, 6). Typically, beta amplitude rebounds to above-baseline levels following a motor response, termed post-movement beta rebound (PMBR). Previously, we found that in patients with schizophrenia recruited in a stable state of their illness, PMBR was attenuated compared with control subjects, with greater attenuation in those with more severe disorganization symptoms and greater impairment of cognitive and role function (2,5). In healthy control participants, we found that PMBR attenuation was associated with higher schizotypal personality scores, most strongly with subscale scores on a factor reflecting subclinical disorganization traits (7), suggesting that this association is not an artifact of medication.

In psychosis, disorganization is predictive of persisting illness and impairments of cognition and role function (8, 9, 10, 11) and has been proposed as a marker for a core deficit in psychosis (5,11). Understanding the nature of PMBR and of its attenuation in psychosis therefore may illuminate the mechanisms underlying persisting illness.

Converging evidence (12, 13, 14) indicates that PMBR signals low prediction error and thus confirmation of the current forward model conceptualized by Wolpert and Ghahramani (15) as an internal representation of the predicted state of the system after an intended movement. Prediction errors are likely to be dopamine mediated (16). Cao and Hu (13) proposed specifically that beta rebound magnitude indexes the extent to which the current forward model matches the sensory reafference following movement completion. They noted that this is consistent with the proposal by Engel and Fries (17) that beta is a top-down signal to actively maintain the status quo.

Engel and Fries (17) proposed that pathological dopamine depletion in Parkinson’s disease enhances beta oscillations, leading to “inability to modify their status quo.” They extended this status quo concept from the sensorimotor domain to the perceptual-cognitive domain, where they proposed that top-down beta signaling overrides potential effects of novel or unexpected external events.

PMBR is normally observed in trial-averaged data as a continuous period of elevated beta amplitude. Jones *et al.* (18,19) reviewed studies showing that in single-trial data, including data on post-movement beta power (20) and working memory (21), beta oscillations manifest as transient stereotyped bursts, with a typical duration of less than 150 ms, and a stereotypical wave form. Smooth power changes in trial-averaged data actually reflect modulation of burst probabilities (19).

Consistent with the Engel and Fries (17) model, Shin *et al.* (22) demonstrated that a transient beta burst just prior to a liminal stimulus reduced the probability of stimulus detection. In primates, Lundqvist *et al.* (21) found higher beta bursting rates in content-encoding neurons during memory maintenance, potentially protecting encoded content from interference by new stimuli. Kornblith *et al.* (23) found similar effects in trial-averaged data.

Going beyond the concept of beta as a status quo–maintaining signal, Spitzer and Haegens (24) proposed that beta bursts play an active role in information processing. They proposed that during memory maintenance, task-relevant information is encoded in content-specific neuronal assemblies, latently maintained by short-term synaptic facilitation, and that beta bursts serve to endogenously reactivate these assemblies. It is plausible that a similar mechanism is involved in reactivating a latently represented forward model when it matches the sensory reafference signal following a movement (13).

Concurrent electroencephalography (EEG)/functional magnetic resonance imaging (fMRI) has the potential to reveal the spatial distribution of brain networks associated with beta bursts. Although conceptually related, the hypothesis that beta bursts play an active role in restoring encoded content would seem to make different predictions from the hypothesis that beta bursts play an inhibitory role in preserving encoded content from interference by extraneous stimuli. If beta bursts serve to reactivate latent content-specific representations, their blood oxygen level–dependent (BOLD) correlates should reveal those content networks and support the findings of Laufs *et al.* (25) of an association between beta and networks implicated in spontaneous cognitive operations. However, if beta bursts serve to inhibit interference from external events, their BOLD correlates might be expected to lie in task-negative networks, such as the default mode network (DMN), that are switched off during tasks requiring attention to external stimuli (26). This would be consistent with Mantini *et al.*’s (27) resting-state study showing a positive correlation between beta power and DMN activation.

Such a study could clarify the mechanisms underlying PMBR attenuation in psychosis. Attenuated PMBR suggests less consistent modulation of beta burst probability in service of task demands. Possibly, brain activity reactivated by a beta burst is less consistently related to task content, reducing correlations between beta bursts and BOLD signals in any one region. Alternatively, beta bursts may induce less synchrony in patients, making neural recruitment less efficient (28,29) and increasing energy demands, leading to greater and/or more extensive beta burst–related BOLD effects.

We collected concurrent EEG/fMRI data from healthy control participants and patients with psychosis to address these questions. Because we were interested in how PMBR abnormalities relate to symptom and outcome heterogeneity within psychosis, we recruited patients with diagnoses of schizophrenia or schizoaffective disorder (here, collectively referred to as Sz) and bipolar disorder (BD). Substantial evidence indicates overlap in the etiology and pathophysiology of these psychoses (30, 31, 32, 33) and that the different diagnoses represent a continuum of impairment, with evidence for more persistent functional impairment (34) and more extensive brain structural abnormality in schizophrenia (32,33).

Data were acquired during performance of a working memory task under three conditions of load as well as during intervening rest periods. We predicted that the BOLD correlates of beta bursts would correspond to cognitive/sensorimotor networks relevant to the task (either task positive or task negative) and that the strength and/or regional extent of these correlates would be abnormal in patients. We also sought to confirm that PMBR, when quantified as increased probability of a beta burst, is attenuated in patients with psychosis and that the degree of attenuation correlates with persisting symptoms and impairments.

## Methods and Materials

### Participant Details

The study was approved by the National Research Ethics Committee (Derbyshire, United Kingdom). All participants provided written informed consent. Patients with a psychotic disorder (BD or Sz) were referred to the study by community-based mental health teams in Nottinghamshire and Leicestershire, United Kingdom. Case note review and standardized symptom assessments from the Signs and Symptoms in Psychotic Illness scale (35) were used in clinical consensus meetings of 3 or more research psychiatrists and psychologists to establish a consensus diagnosis according to DSM-IV criteria (36) following the procedure of Leckman *et al.* (37). Patients needed to be in a stable phase of their illness. Healthy control participants were recruited by means of advertisements in the local community targeted to achieve matching with the patient groups on age, gender, and parental socioeconomic classification (5-band version) (38). (For full details of inclusion and exclusion criteria, see Inclusion and Exclusion Criteria in the Supplement.)

We included data from 78 participants, consisting of 32 patients with a diagnosis of Sz (26 men and 6 women), 16 patients with a diagnosis of BD (9 men and 7 women), and 30 healthy control participants (21 men and 9 women). The mean age of the sample was 33.9 years (SD = 9.5). None of the three groups differed significantly in age (*F* < 1), gender (χ^2^_2_ = 3.370, *p =* .185), handedness (proportion right-handed) *(*χ^2^_2_ = 0.440, *p =* .802), or parental socioeconomic class (5-band version) (χ^2^_8_ = 8.047, *p =* .429).

### Clinical Measures

Cognitive function was assessed using a customized written and oral Digit Symbol Substitution Test (DSST) (39,40). For patients, DSM-IV-based Global Assessment of Functioning (GAF) and Social and Occupational Functioning Assessment Scale scores were assigned based on the Signs and Symptoms in Psychotic Illness interview (35). To assess persistence of symptoms, symptom clusters from the Signs and Symptoms in Psychotic Illness scale representing reality distortion, disorganization, psychomotor poverty, psychomotor excitation, and depression were scored from clinic notes for persistence on a scale from 0 to 6, where a score of 0 indicates an absence of symptoms and 6 indicates continuous presence of at least some symptoms (41) (for methods, see Symptom Persistence Measures in the Supplement).

### Behavioral Task

During scanning, participants performed two runs of an n-back working memory task coded in Presentation software (42). Each run contained seven task blocks interspersed with 30-second rest intervals. Each task block consisted of 0-back, 1-back, and 2-back sub-blocks presented in a random order and separated by 10-second rest intervals. In each sub-block, 15 letters (including 4 targets requiring a right index finger button press) were projected sequentially through goggles at 2-second intervals. A total of 10 different letter stimuli were used. In the 0-back condition, the target was the letter X. In the 1-back condition, the target was any letter that matched the immediately preceding letter. In the 2-back condition, the target was any letter that matched the letter presented two trials previously. Working memory load therefore increased monotonically from the 0-back condition to the 2-back condition.

### EEG Acquisition and Preprocessing

EEG data from 31 electrodes were recorded using an MR-compatible apparatus concurrently with 3T fMRI acquisition. Recordings from each participant and run underwent preprocessing, including correction of gradient and cardioballistic artifacts, followed by independent component analysis (ICA) to remove residual artifacts as well as eye movement and single-channel noise artifacts. Data epochs from each participant were then filtered into the beta frequency band, concatenated, and submitted to a group ICA to identify a single representative ICA component that most clearly represented beta band brain activity. (For details of EEG recording and EEG preprocessing, see EEG Acquisition and Pre-processing in the Supplement.)

**Figure 1:**
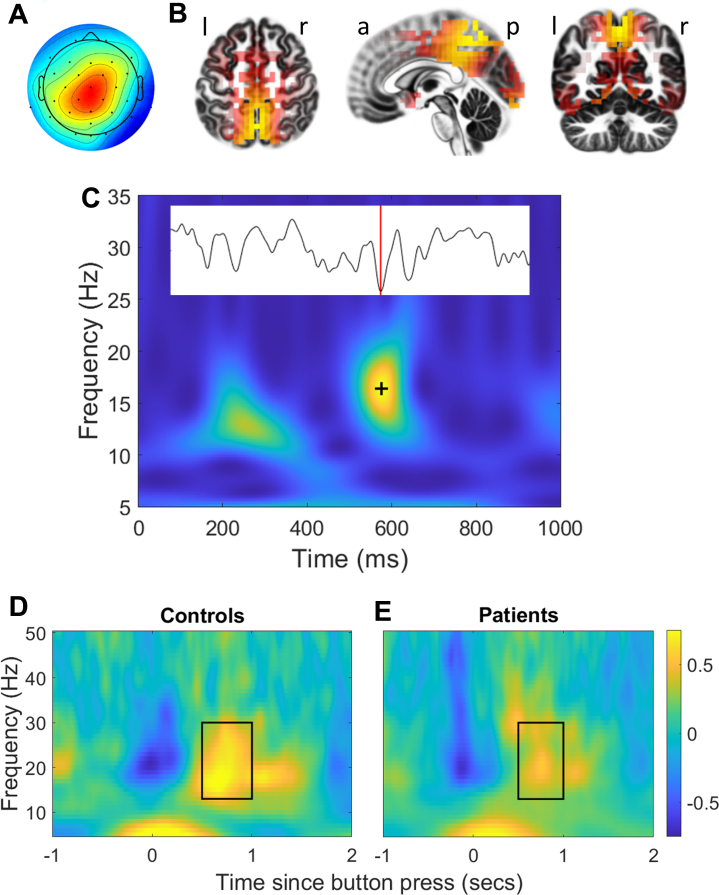
Group independent component analysis (ICA) component selected for identifying beta bursts, example of a beta burst, and average time–frequency spectrograms showing mean movement-related beta modulation. **(A)** Topography of the group ICA component chosen to derive neural activity time courses for identifying beta bursts in the continuous electroencephalography data of individual participants. **(B)** Standardized low-resolution brain electromagnetic tomography source analysis (57) of the chosen group ICA component, plotted on the Montreal Neurological Institute 152 brain (58) using LORETA-Key software (http://www.uzh.ch/keyinst/loreta.htm). **(C)** Time–frequency spectrogram showing a beta event that exceeded the selection threshold (black plus sign). Yellow and blue represent areas of high and low spectral power, respectively. The inset image shows the corresponding component time course, with the plus location marked by a vertical red line. The abscissa is arbitrarily set to start at zero for this image. The spectrogram was constructed using 0.1-Hz bins, while the analysis was conducted using 1-Hz bins. **(D, E)** Time–frequency spectrograms showing the traditionally measured post-movement beta rebound for control participants **(D)** and patients **(E)**, computed by averaging time–frequency spectrograms across epochs, relative to the time of a motor response. Colors represent power in decibels relative to power in the −3- to −1.5-second baseline window used to calculate post-movement beta rebound in this article. The black rectangle encases the post-movement beta rebound window used in this study. a, anterior; l, left; p, posterior; r, right.

The ICA weights of the selected group component were applied to the continuous, preprocessed, broadband-filtered data from each participant and run to derive continuous time courses of neural activity. For each time course, a time–frequency decomposition was computed using 5-cycle Morlet wavelets with frequencies at 1-Hz intervals from 1 to 40 Hz using the mfeeg toolbox (43). The resulting time–frequency spectrograms were filtered with a two-dimensional Gaussian filter (standard deviations: 1 Hz/6 ms), and peaks were identified using image dilatation with a 5-by-5 structuring element consisting of ones with a center value of zero (implementation: Tony Fast; https://gist.github.com/tonyfast/d7f6212f86ee004a4d2b). Peak values less than 6 times the median power across all time points at the peak frequency were excluded (22). An example of a burst that exceeded this threshold is shown in Figure 1C. MATLAB code (The MathWorks, Inc.) implementing the above analysis steps is available under a General Public License (version 2.0) (https://github.com/pmbriley/beta_bursts). The times of the selected peaks were used as event times in the fMRI analysis.

Mean beta burst rates were calculated for each participant and experimental condition and were compared using analyses of variance implemented in SPSS Statistics (version 25) (IBM Corp.). Analyses of variance were also used to compare PMBR values, with PMBR calculated as the mean beta burst rate in windows extending from 0.5 to 1 second after button presses minus the rate in baseline windows extending from 3 to 1.5 seconds before button presses. Greenhouse–Geisser adjustments to degrees of freedom were made where Mauchly’s nonsphericity test was significant. Nonparametric statistics were used where other assumptions for parametric statistics were not met.

### fMRI Acquisition and Analysis

3T BOLD fMRI data were acquired concurrently with the EEG. SPM12 (44) was used for fMRI analyses. First-level general linear models were constructed for each participant, incorporating both data runs. Design matrices included 0-back, 1-back, and 2-back sub-blocks modeled as boxcars convolved with the SPM canonical hemodynamic response function as well as motor responses and beta bursts modeled as impulses convolved with the same function. First-level images for the beta event above baseline contrasts were entered into second-level random effects analyses. (For full details of fMRI recording and preprocessing, including details of a control analysis using fake events in place of beta bursts, see fMRI Acquisition and Pre-processing in the Supplement.)

The preceding analyses identified 5 significant activation clusters positively associated with beta bursts. For each cluster, a spherical region of interest (ROI) with a 1-cm radius was constructed, centered on the voxel with peak significance. Regression coefficients for the beta burst contrast were obtained for each participant and ROI and were entered into an analysis of variance to compare across brain areas and participant groups.

## Results

### Clinical Measures and Behavioral Results

Group characteristics and statistical tests of group differences are detailed in Table 1. Notably, patients were significantly more impaired on the DSST (39) than healthy control participants. Patients with Sz were significantly more impaired than patients with BD on both the DSST and on the GAF (36). Patients with Sz and BD differed in their profiles of persistent symptoms, but not significantly in persistence of disorganization.

**Table 1:** Descriptive and Comparative Statistics for Clinical Measures and Measures of Task Performance

Patients With Sz Versus Patients With BD							
General	Mean (SD)				*t* (*df*)	Significance	Effect Size *d*
	Sz (*n* = 32)	BD (*n* = 16)					
Duration of Illness	9.1 (7.7)	11.9 (9.0)			1.13 (46)	.265	–
Age at Onset of Illness	23.8 (5.2)	24.0 (5.6)			0.15 (46)	.878	–
GAF	48.9 (11.8)	59.4 (13.2)			2.79 (46)	.008	0.84
DSST	41.9 (10.3)	49.8 (9.0)			2.62 (46)	.021	0.82
DDD Antipsychotic	1.32 (1.20)	0.50 (0.52)			3.33 (45.5)	.002	1.90
DDD Mood Stabilizer	0.01 (0.05)	0.98 (0.62)			6.22 (15.1)	.000	1.91
DDD Antidepressant	0.30 (0.55)	0.09 (0.38)			1.50 (41.5)	.141	–

Symptom Persistence	Median (Min:Max)		Mean Rank (High to Low; *N* = 45)		Mann–Whitney *U*	Exact Significance	Effect Size *r*
	Sz (*n* = 32)	BD (*n* = 16)	Sz	BD			
Reality Distortion	4 (1:6)	1.5 (0:3)	30.8	12.0	56.0	<.001	0.65
Disorganization	1 (0:6)	2 (0:2)	24.3	24.8	251.0	.887	–
Psychomotor Poverty	1 (0:6)	0.5 (0:4)	25.3	22.8	229.5	.544	–
Psychomotor Excitation	0 (0:2)	2 (1:4)	17.6	38.4	34.0	<.001	0.75
Depression	1.5 (0:5)	2 (0:5)	23.1	27.4	210.0	.311	–

Healthy Control Participants Versus Patients With Psychosis							
n-Back Performance	Median (IQR)		Mean Rank (High to Low; *N* =78)		Mann–Whitney *U*	Exact Significance	Effect Size *r*
	C (*n* = 30)	P (*n* = 48)	C	P			
Accuracy 0-Back	100.0 (0)	100.0 (1)	30.7	45	456.0	.001	0.37
Accuracy 1-Back	100.0 (1)	99.1 (3)	31.6	44.5	482.0	.012	0.28
Accuracy 2-Back	97.9 (5)	95.2 (6)	31.9	44.3	490.5	.018	0.27
Median RT 0-Back	433 (74)	464 (68)	47.1	34.8	493.0	.019	0.37
Median RT 1-Back	447 (100)	527 (175)	46.5	35.1	510.5	.031	0.28
Median RT 2-Back	539 (177)	629 (213)	47.0	34.8	495.0	.020	0.27

	Mean (SD)						
Cognition	C (*n* = 30)	P (*n* = 48)			*t* (*df*)	Significance	Effect Size *d*
DSST	58.1 (9.4)	44.5 (10.5)			5.75 (76)	<.001	0.84

Comparisons between patient groups are shown in the upper panel, and comparisons between healthy control participants and patients with psychosis are shown in the lower panel.

BD, bipolar disorder; C, control; DDD, defined daily dose; DSST, Digit Symbol Substitution Test; GAF, Global Assessment of Functioning; IQR, interquartile range; P, all patients; RT, reaction time; Sz, schizophrenia or schizoaffective disorder.

**Figure 2:**
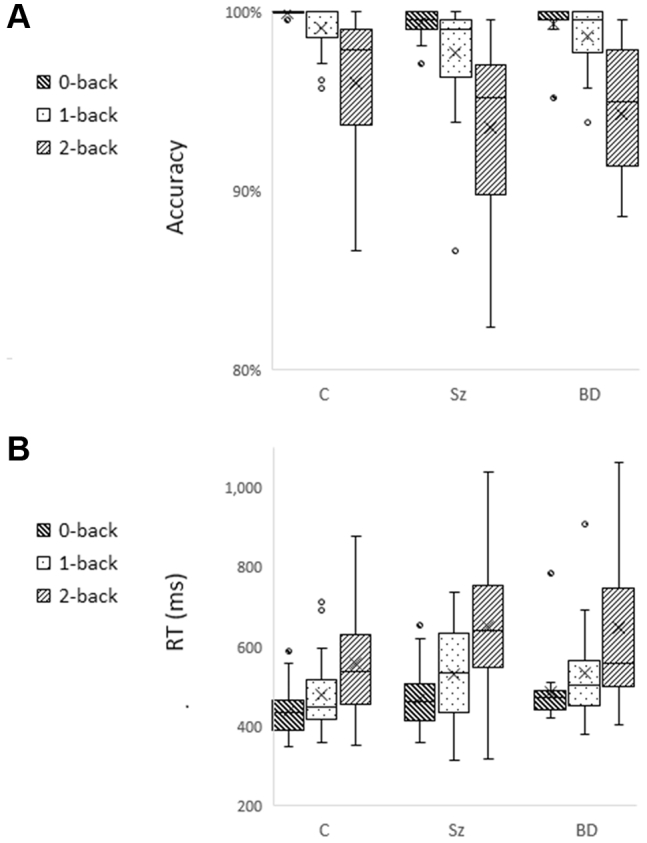
Performance by each group on the n-back task. No participant made any errors of commission, and the target detection rate was significantly above chance (*p* < .001) for all participants. Nonetheless, all groups missed more targets at higher loads than at lower loads **(A)**, and patients were overall significantly less accurate than healthy control participants. Reaction times (RTs) **(B)** were also longer at higher loads, and patients were significantly slower overall than healthy control participants. Upper and lower bounds of each box denote interquartile range, horizontal line denotes median, X denotes mean, whiskers denote range if within 1.5 × interquartile range, and datapoints outside this range are shown as circles. BD, bipolar disorder; C, control; Sz, schizophrenia or schizoaffective disorder.

On the n-back task, all participants performed significantly above chance at all levels of load (*p* < .001), and none made any errors of commission. Overall, performance declined with increasing load (Figure 2); accuracy declined, χ^2^_2_ = 120, *p* < .001, Cramer’s *V* = 0.88, and reaction time increased, χ^2^_2_ = 84, *p* < .001, Cramer’s *V* = 0.71 (Friedman’s exact test). Between-group performance findings are summarized in Table 1. Patients were significantly slower and less accurate than healthy control participants. The two patient groups did not differ significantly on any performance measure.

### Post-movement Beta Rebound

**Figure 3:**
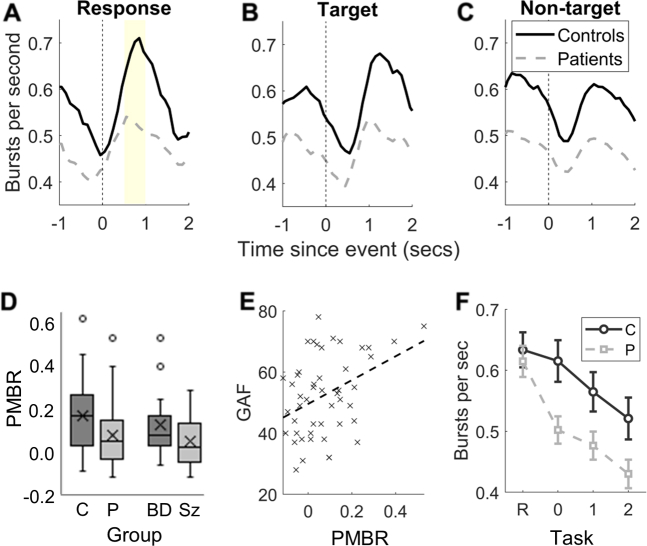
Relationships between beta burst rate and timings of responses or stimuli as well as relationships with clinical group, Global Assessment of Functioning (GAF), and task difficulty. **(A–C)** Mean beta bursts per second calculated in 500-ms sliding windows for control participants (solid black lines) and patients (dashed gray lines) time locked to motor responses **(A)**, target stimuli **(B)**, and nontarget stimuli **(C)**. The shaded region in **(A)** indicates the post-movement beta rebound (PMBR) window. **(D)** Boxplots showing distributions of PMBR (increase in burst probability following a motor response relative to the baseline window) for control participants (C) vs. patients (P) and for patients with bipolar disorder (BD) vs. patients with schizophrenia or schizoaffective disorder (Sz) (collapsed across task conditions). Upper and lower bounds of each box denote interquartile range, horizontal line denotes median, X denotes mean, whiskers denote range if within 1.5 × interquartile range, and datapoints outside this range are shown as circles. **(E)** GAF score plotted against PMBR (collapsed across task conditions). Each data point is a single patient. The dashed line indicates the line of best fit. **(F)** Mean beta burst rate for control participants and patients for rest (R), 0-back, 1-back, and 2-back conditions. Error bars denote ±1 SEM.

As with traditionally measured PMBR, following a motor response, beta burst rate rebounded above baseline, reaching a maximum approximately 0.5 to 1 second post response (Figure 3A). These effects were greater in control participants than in patients (see also Figure 1D, E for time–frequency spectrograms illustrating event-related trial-averaged power). A time-shifted version of this pattern is seen when burst rate is calculated relative to the onset of target stimuli (Figure 3B). All stimuli were initially followed by a reduction in beta burst rate, but for nontarget stimuli this was followed by a return to baseline burst rate only, with no above-baseline rebound (Figure 3C).

There was no significant modulation of PMBR by memory load, *F*_2,152_ = 0.929, *p* = .397. PMBR differed significantly between groups, *F*_2,75_ = 5.201, *p* = .008, η_p_^2^ = 0.12. Planned orthogonal contrasts confirmed that PMBR was significantly attenuated in patients relative to control participants (*p* = .026). The mean PMBR in patients with BD was intermediate between patients with Sz and control participants, but the difference between the patient groups did not reach significance (*p* = .085) (Figure 3D).

PMBR was associated with greater impairments in overall functioning as measured by the GAF, *ρ*_48_ = 0.318, *p* = .028 (Figure 3E), and by the Social and Occupational Functioning Assessment Scale, *ρ*_48_ = 0.325, *p* = .024. PMBR was associated with poorer performance on the DSST, *ρ*_48_ = 0.399, *p* = .005, and greater persistence of disorganization symptoms, *ρ*_48_ = −0.325, *p* = .024, but not with persistence of other symptoms. These associations remained significant after controlling for age, gender, and defined daily dose of antipsychotic, antidepressant, and mood stabilizer medication in a hierarchical regression model, and none of the covariates was a significant predictor of PMBR.

To test the hypothesis that attenuated PMBR is associated with a core deficit (11) reflected in variance shared among these clinical, functional, and cognitive measures, we conducted a multivariate general linear model with PMBR together with the other covariates as predictor variables. Dependent variables were GAF, DSST, and disorganization scores [we omitted the Social and Occupational Functioning Assessment Scale because it correlated highly with GAF scores, *ρ*_48_ = 0.702, *p* < .001]. PMBR was a significant multivariate predictor of this group of variables, *F*_3,44_ = 5.160, *p* = .004, η_p_^2^ = 0.260.

### Modulation of Beta Burst Rate by Working Memory Load

Beta burst rate during rest periods (no working memory load) and for the task blocks in each n-back condition decreased monotonically with increasing working memory load, *F*_3,228_ = 33.573, *p* < .001, η_p_^2^ = 0.31 (Figure 3F). This reduction in beta burst rate with load was significantly greater in patients than in control participants, *F*_3,228_ = 3.532, *p* = .016, η_p_^2^ = 0.05. Patients’ beta burst rates were similar to those of control participants in the rest condition (*p* = .632) but were significantly lower than those of control participants in each of the n-back task conditions (0-back: *p* = .005, *d* = 0.66; 1-back: *p* = .027, *d* = 0.52; 2-back: *p* = .023, *d* = 0.52).

### BOLD Correlates of Beta Bursts

#### All Beta Bursts

**Figure 4:**
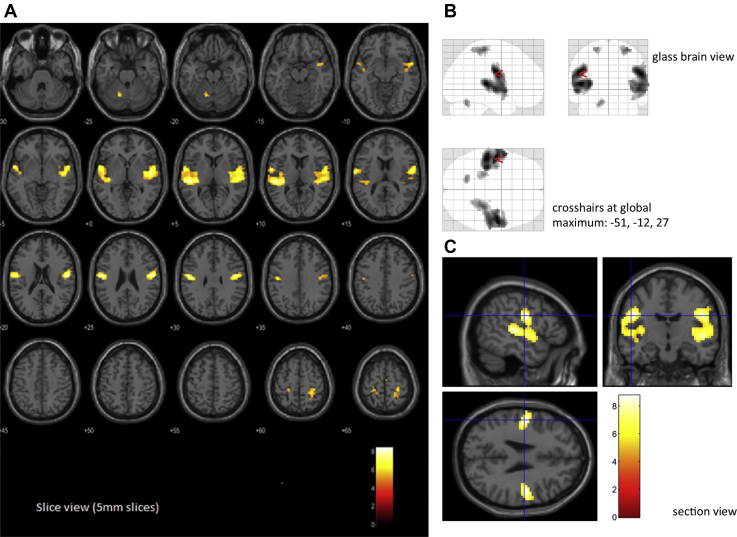
Clusters (*k* ≥ 20) showing significant increases in blood oxygen level–dependent activity associated with beta bursts (cluster threshold *p* < .05, familywise error corrected; voxel threshold *p* < .05, false discovery rate corrected). **(A)** Axial 5-mm slice view overlaid on the SPM single-subject brain. **(B)** Clusters shown on the SPM glass brain in three orthogonal planes. **(C)** Orthogonal sections through the global maximum (Montreal Neurological Institute: x = −51, y = −12, z = 27) overlaid on the SPM single-subject brain.

Figure 4 shows areas of significantly increased BOLD activity associated with beta bursts after controlling for task block and motor responses with separate regressors. With a voxel significance threshold of *p* < .05, false discovery rate corrected, and a minimum cluster size of 20, five significant clusters of activation met a cluster significance threshold of *p* < .05, familywise error corrected. The largest cluster in each hemisphere included inferolateral precentral/postcentral gyrus (mouth and pharynx areas of the motor/somatosensory homunculus) as well as the superior and transverse temporal gyri (Table 2). We refer to these clusters as the sensorimotor-verbal (SM-verbal) clusters (peak significant voxels; left: x = −51, y = −12, z = 27; right: x = 54, y = −9, z = 24). There were also clusters in the left and right superior precentral/postcentral gyrus (in the vicinity of the hand area of the motor/somatosensory homunculus). We refer to these clusters as the sensorimotor-manual (SM-manual) clusters (peak significant voxels; left: x = −24, y = −33, z = 63; right: x = 30, y = −30, z = 63). Finally, there was a cluster in the left cerebellum (peak significant voxels: x = −15, y = −63, z = −24). In a sensitivity analysis, we excluded motor responses from the fMRI design matrices; this had little impact on the clusters identified (see BOLD Correlates of Beta-Bursts Without Motor Response Regressors in the Supplement). There were no clusters with significantly decreased BOLD signal associated with beta bursts in this analysis. This was because the areas negatively correlated with beta bursts differed between task and rest blocks (discussed below).

**Table 2:** The Five Clusters of Blood Oxygen Level–Dependent Activity Positively Associated With Beta Bursts

Cluster Name	Peak Significant Voxel (MNI Coordinates)	AAL Atlas Regions	% of Cluster
Left Sensorimotor-Verbal	x = −51, y = −12, z = 27	Superior temporal gyrus	42%
		Postcentral gyrus	19%
		Rolandic operculum	12%
		Heschl’s gyrus	7%
		Insula	6%
		Precentral gyrus	4%
		Middle temporal gyrus	2%
Right Sensorimotor-Verbal	x = 54, y = −9, z = 24	Superior temporal gyrus	33%
		Rolandic operculum	18%
		Insula	13%
		Postcentral gyrus	12%
		Heschl’s gyrus	7%
		Precentral gyrus	3%
Left Sensorimotor-Manual	x = −24, y = −33, z = 63	Postcentral gyrus	89%
		Precentral gyrus	7%
		Precuneus	4%
Right Sensorimotor-Manual	x = 30, y = −30, z = 63	Postcentral gyrus	78%
		Precentral gyrus	13%
		Superior parietal gyrus	8%
Left Cerebellum	x = −15, y = −63, z = −24	Cerebellum 6	95%
		Cerebellum 4/5	5%

Clusters were derived from the maps shown in Figure 4 (minimum cluster size of 20 voxels). For each cluster, the table gives coordinates of the peak significant voxel (millimeters in Montreal Neurological Institute [MNI] space), the Automated Anatomical Labeling (AAL) atlas (59) regions covered, and the percentage of the cluster attributed to each AAL region. AAL regions accounting for less than 2% of each cluster have been omitted (hence some values do not sum to 100%), and the temporal pole of the superior temporal gyrus has been included in the superior temporal gyrus percentages.

#### Beta Burst BOLD Signals During Task and Rest

**Figure 5:**
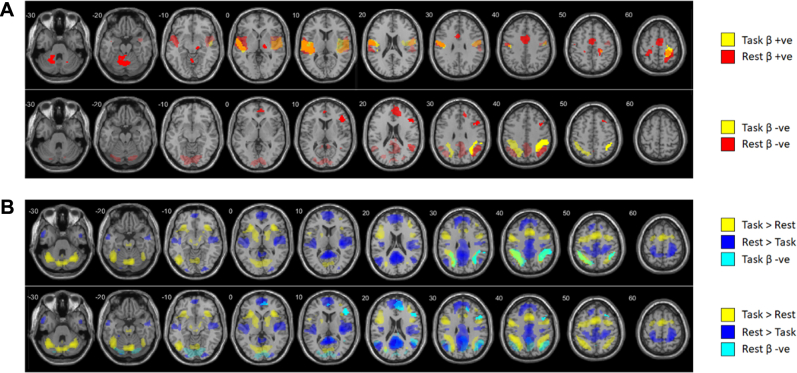
Beta burst effects during task and rest compared with the correlates of task and rest blocks. All clusters shown are of voxels significant at *p* < .001, uncorrected, in clusters significant at *p* < .05, familywise error corrected. **(A)** Positive and negative correlates of beta bursts. Top row: Positive correlates of task beta bursts (Task β+ve) and rest beta bursts (Rest β+ve). Bottom row: Negative correlates of task beta bursts (Task β−ve) and rest beta bursts (Rest β−ve). Beta bursts activated similar regions whether produced during task or rest, but they suppressed different regions. **(B)** Negative correlates of task beta bursts (top) and rest beta bursts (bottom) overlaid on correlates of task block (Task > Rest and Rest > Task). Beta bursts produced during task suppressed regions otherwise more active during task, while beta bursts produced during rest suppressed regions otherwise more active during rest.

We then analyzed the BOLD correlates of beta bursts produced during task blocks and those produced during rest periods separately. Positive BOLD correlates of task beta bursts and rest beta bursts were similar (Figure 5A, top row), including activation in the SM-verbal and SM-manual clusters, although BOLD correlates of beta bursts during rest were more positive in motor regions, likely due to the separate modeling of motor responses during task blocks.

Strikingly, however, there were strong negative BOLD correlates of beta bursts in this analysis, and these correlates differed between task and rest (Figure 5A, bottom row). Specifically, regions negatively correlated with beta bursts tended to overlap with regions more active during the current condition; beta bursts produced during task blocks were associated with reduced activity in regions otherwise more active during the task (Figure 5B, top row), while beta bursts produced during rest were associated with reduced activity in regions otherwise more active during rest (Figure 5B, bottom row). In short, beta bursts were associated with phasic inhibition of areas that were currently tonically active (see BOLD Correlates of Beta-Bursts Relative to Task and Motor Responses in the Supplement).

#### Patient Abnormalities in Beta Burst–Related BOLD Signal

**Figure 6:**
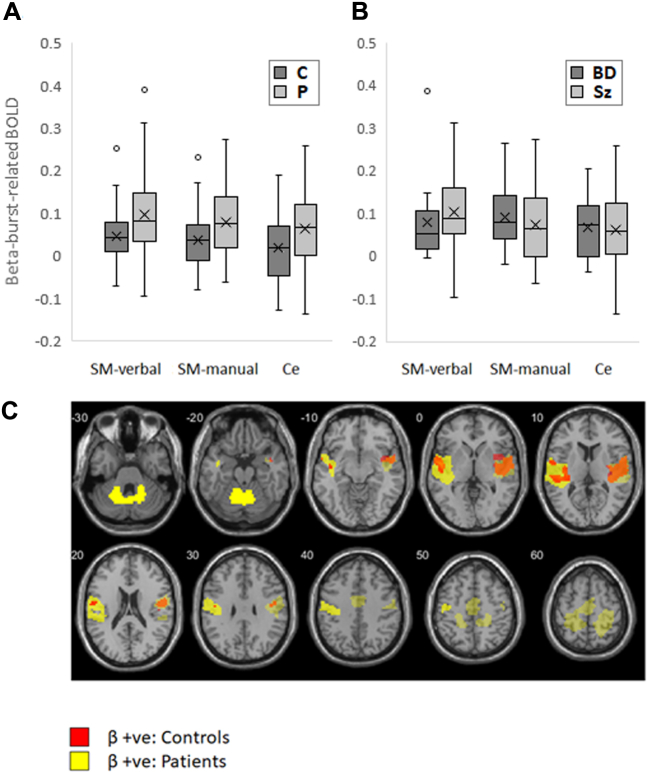
Boxplots showing distribution of blood oxygen level–dependent (BOLD) activation associated with beta bursts within sensorimotor-verbal (SM-verbal), sensorimotor-manual (SM-manual), and left cerebellar (Ce) regions of interest. Upper and lower bounds of each box denote interquartile range, horizontal line denotes median, X denotes mean, whiskers denote range if within 1.5 × interquartile range, and datapoints outside this range are shown as circles. **(A)** Control participants (C) vs. patients (P). **(B)** Patients with bipolar disorder (BD) vs. patients with schizophrenia or schizoaffective disorder (Sz). **(C)** Significant clusters of positive beta burst–related activity (β+ve) in patients (yellow) compared with control participants (red). Areas of overlap are shown in orange. Activation by beta bursts was not only stronger in patients but also more extensive.

In three ROIs centered on the three clusters that had shown significant beta burst–related activation (SM-verbal, SM-manual, and cerebellar; collapsed across hemispheres in the first two cases) (Figure 4 and Table 2), beta burst activation was significantly elevated in patients compared with control participants, *F*_1,76_ = 11.178, *p* = .001, η_p_^2^ = 0.13 (Figure 6A), with no significant difference between ROIs in the degree of elevation, *F*_2,152_ = 0.051, *p* = .950. There were no significant differences between the Sz and BD groups (Figure 6B), *F*_1,46_ = 0.000, *p* = .986. Whole-brain analyses of beta burst–associated activations in each group separately revealed more extensive clusters in patients than in control participants (Figure 6C), and a direct group comparison revealed two significant clusters of increased activation in patients: a medial cluster that included regions of the salience network (45), and a cluster in the left sensorimotor cortex.

## Discussion

Our data show that transient beta bursts have clear, anatomically plausible BOLD correlates. Our data also support the conceptualization of PMBR as an increase in beta burst probability following a movement. Our findings strengthen the evidence for attenuated PMBR in patients with psychosis in a stable stage of their illness. They also strengthen the evidence for a specific link between attenuated PMBR and a core deficit associated with persisting disorganization symptoms, cognitive impairment, and poor outcome (8, 9, 10, 11) regardless of specific diagnosis. Our findings are consistent with the hypothesis that Sz and BD lie on a continuum, with more severe persisting impairments in Sz.

We hypothesized that if beta bursts play an active role in reactivating latent, content-specific representations (24), the positive BOLD correlates of beta bursts would include brain networks associated with encoded content. We observed BOLD correlates of beta bursts in bilateral SM-verbal areas as well as areas implicated in a motor response. The SM-verbal areas include areas implicated in overt and covert speech and in the processing of speech sounds and language (46,47). Because the content in the n-back task was letters, it is plausible that these areas represent reactivated phonological encoding of the letter names. However, similar beta burst activation clusters were seen during rest periods. Possibly, spontaneous recall of the letter stimuli so recently presented may have been predominant in the content reactivated by beta bursts during these rest intervals. Alternatively, it may be that this pattern of activation during rest reflects reactivation of sensorimotor representations of verbal thought unrelated to the n-back task.

As an alternative hypothesis, we predicted that if beta bursts serve to inhibit bottom-up interference from external events, positive BOLD correlates of beta bursts would lie in regions of the DMN, consistent with Mantini *et al.* (27). We did not find any positive association between beta bursts and DMN regions. Instead, beta bursts were associated with a phasic reduction of BOLD signal in current tonically active networks: task-positive regions during task periods and task-negative regions during rest periods. However, this pattern would still be consistent with the idea that beta bursts serve to protect encoded content from interference from competing signals, as proposed by Lundqvist *et al.* (21), if we consider that interference could arise both from external stimuli, as during task periods, and from competing spontaneous mental events, as during rest.

Despite an attenuated increase in beta burst rate in the PMBR window and reduced overall beta burst rate during the task, patients showed stronger and more widespread beta burst–related BOLD signal. This could reflect an inefficiency of beta burst–mediated cortical synchrony that is more energy demanding and/or necessitates more extensive neural recruitment (28,29). Indeed, an inefficiency hypothesis is consistent with the conceptualization of schizophrenia as a disconnection syndrome (48, 49, 50, 51). A related but more specific explanation may be that latent content-specific representations reactivated by beta bursts (24) may be less precisely specified in psychosis, potentially giving rise to working memory impairments (52,53) and to the loosening of associations characteristic of disorganization.

The finding that beta bursts during the n-back task not only were associated with activation of plausibly content-specific brain areas but also increased in rate during the PMBR window supports the proposal that PMBR itself represents the reactivation of task-relevant latent representations. This would be consistent with the proposal by Cao and Hu (13) that beta rebound signals a match between the internally represented forward model and the sensory reafference signal following completion of a movement. It is also consistent with our speculation that post-movement beta bursts may reactivate the internally represented forward model by the mechanism proposed by Spitzer and Haegens (24) for reactivating working memory content.

Jenkinson and Brown (54) proposed that beta oscillations are modulated by net dopamine levels, which in turn are modulated by salient and/or unpredicted stimuli (16). These trigger dopamine release, suppressing beta oscillations and increasing motor readiness. This mechanism could account for both the well-attested phenomenon of stimulus-related reductions in beta power (55) and the poststimulus reductions in beta burst rate we observed following both target trials (Figure 3B) and nontarget trials (Figure 3C). Note that in the n-back task, both targets and nontargets are salient and unpredictable as to content. In this reading, dopamine-mediated suppression of beta bursts during stimulus presentation may facilitate encoding while suppressing interference from reactivation of other latently maintained encoded representations. Conversely, when a sensory afferent signal matches the latently encoded forward model following a correct response, net dopamine may fall, allowing a beta burst to reactivate the forward model as a representation of the current status quo.

Modulation of dopamine by task stimuli may also account for the greater reduction of beta burst rate with greater load; as load increases, so do both the coding burden and the need to suppress inadvertent reactivation of current latently stored representations of previous stimuli. If so, the greater reduction of beta burst rate with increasing load in patients may reflect reduced capacity to predict the onset of stimuli and/or less efficient encoding processes.

Although beta burst rate in unmedicated patients might be suppressed by clinically elevated dopamine levels, most of our study patients were medicated and in a stable state, and their defined daily dose of dopamine antagonist medication was not a significant predictor of PMBR. An alternative hypothesis is that abnormalities in the mechanisms by which beta bursts coordinate reactivation of contextually appropriate content can manifest as disorganization, working memory deficits (53), and inaccurate forward models (56). Under stress, these may lead to a surfeit of dopamine-mediated error signals that precipitate the dopamine dysregulation of acute psychosis as well as reflect the core deficit (5,11) associated with persisting impairment.
