# Supplementary material for: Regional Brain Correlates of Beta Bursts in Health and Psychosis: A Concurrent Electroencephalography and Functional Magnetic Resonance Imaging Study
Source: Biol Psychiatry Cogn Neurosci Neuroimaging. 2021 Dec;6(12):1145–56. doi: 10.1016/j.bpsc.2020.10.018 (PMC8648891; doi:10.1016/j.bpsc.2020.10.018)
Supplement: Supplementary Materials [file mmc1.pdf]

Regional Brain Correlates of Beta Bursts in Health and Psychosis: A Concurrent Electroencephalography and Functional Magnetic Resonance Imaging Study

Supplementary Information

SUPPLEMENTAL TEXT

Inclusion and exclusion criteria

Both patients and healthy control participants had to: be over eighteen and under fifty years old; have an estimated IQ of at least 70 (Quick Test; (1)); have no lifetime history of substance dependence, or harmful substance use in the last six months; have no history of head trauma with more than five minutes loss of consciousness; have no history of substantial neurological disorders. All participants had to meet MR safety criteria.

Patients had to be in a stable phase of their illness as measured by no more than ten points difference in score on the Global Assessment of Functioning scale (GAF) (2) from the scan to six weeks prior, and no change in psychiatric medication during this time. Controls had to have no history of any psychiatric disorder and no family history of psychotic illness.

A total of 112 adult participants were recruited to the study. Technical issues with data acquisition accounted for the loss of 26 datasets (15 patients and 11 healthy controls). Four patients withdrew consent during scanning, and one patient's data was excluded due to a brain anomaly evident on the structural scan. EEG data collected concurrently with the remaining fMRI datasets were assessed for quality by a researcher blind to group membership; three EEG datasets (2 patients, 1 healthy control) were excluded due to large and frequent artefacts remaining in the gradient/cardioballistic-artefact-corrected EEG data.

Symptom persistence measures

Persistence of each of the five clusters of symptoms defined in the Signs and Symptoms of Psychotic Illness (SSPI) scale (3) was assessed via independent retrospective review of case notes by two investigators. The case notes contained descriptions of the patient's mental state and behaviour recorded by psychiatrists providing outpatient care, psychiatrists and social workers performing emergency assessments, psychiatrists providing inpatient care, nursing and other paramedical personnel involved in care and also correspondence between General Practitioners and the mental health team.

Presence of symptoms from each of the five clusters was assessed in each episode of illness. An episode was defined as a period when symptoms were present for at least one week and global mental state, as defined by Global Assessment of Functioning (GAF) (2), being appreciably worse than usual, e.g. more than 10 points drop in GAF score. Episodes that were less than 6 months apart were not counted as separate episodes. For each of the five clusters, presence of symptoms was scored 0 if there was definite absence of symptoms judged from one or more sources, 1 if there was questionable evidence of the presence of symptoms, and 2 if there was definite presence of the symptoms judged from one or more sources within the case record.

An overall persistence score for each cluster was assigned, ranging from 0 (no clinically significant symptoms in the relevant cluster recorded at any time during the illness) to 6 (chronic symptoms from within the cluster with evidence that the majority of symptoms had been continually present ever since the first episode.) Further details of the scoring procedure are provided in the notes for the *Persistence of Signs and Symptoms in Psychotic Illness scoring form* at the end of Supplemental Information. The two investigators achieved an adequate

inter-rater reliability. For individual symptom clusters the Intraclass Correlation Coefficient ranged from 0.71 for Disorganization to 0.93 for Reality Distortion.

EEG acquisition and pre-processing

EEG data were recorded using 31 Ag/AgCl electrodes arranged according to the 10-20 electrode system in an MR-compatible elasticated cap (BrainCap MR, Brain Products, Germany). Signals were amplified, bandpass filtered between 0.1 and 250 Hz, and digitised at a sampling rate of 5 kHz (BrainAmp MR Plus, Brain Products), and stored for further analysis (Brain Vision Recorder, v1.10, Brain Products). EEG and MR scanner clocks were synchronised to facilitate gradient artefact correction. Channels were online referenced to an electrode at FCz. Eye movements were monitored with an electrode below the right eye. Impedances were kept below 15 kΩ throughout recordings.

EEG data for each participant and run underwent the following pre-processing steps, implemented in Matlab (4) using the EEGLAB toolbox (v14.1.2) (5):

1. Gradient artefact correction using the FMRIB plug-in (v1.21) (6,7), with data up-sampling to 20 kHz. The plug-in subtracts a moving-average template of the gradient artefact (the default value of 30 artefacts was used for the moving average), and then fits and subtracts optimal sets of principal components derived from the artefact residuals
2. Band-pass filtering (0.5-45 Hz) and down-sampling to 500 Hz
3. Cardio-ballistic artefact correction using the FMRIB plug-in. The plug-in computes a principal component analysis (PCA) of all the cardio-ballistic artefacts, then subtracts, from each individual artefact, an artefact template created by fitting the first N PCA components to that individual artefact (the default value of N = 4 was used)

4. Visual inspection of data and interpolation of bad channels (required in five datasets, up to two channels interpolated)
5. Re-referencing to average reference
6. Splitting the dataset into five-second epochs; epochs containing extreme values (joint probability limits of ± 3.5 SD) were excluded (mean 9 ± 2.2 epochs excluded per run)
7. Independent Components Analysis (ICA) using the Infomax extended ICA algorithm (8–10). Components clearly representing eye movements (Figure S1: A, B), residual cardio-ballistic artefact (Figure S1C; see also Fig. 4B in Mayeli et al. (11), residual gradient artefact (Figure S1D) or single-channel noise, were identified manually by an experienced rater, using the component topographies, time courses, and correlations with the artefact (eye and VCG) time courses, then rejected. 10 ± 2.8 components were rejected per run.

Following rejection of artefactual components, fifty epochs from each participant were randomly selected to contribute to a group ICA in order to identify a single representative component for deriving continuous time courses of brain activity. To identify components characterised by correlated activity in the beta band, epochs were first filtered into this band (13-30 Hz; note this filtering was solely for the group ICA and was not carried over into subsequent steps). Epochs were concatenated in the time dimension to form a single group dataset and submitted to an ICA with an initial principal component analysis that retained the twenty largest components (in order to account for the reduced rank of the data arising from the preceding per-subject ICA component rejection). The CUDAICA toolbox (12) https://github.com/fraimondo/cudaica, which accelerates the Infomax extended ICA algorithm on dedicated graphics processing units, was used for the group ICA. A single ICA

component was selected that most clearly represented beta-band brain activity (Figure 1A, B) by having low time-course correlations with the artefact channels and a source distribution typical of that previously reported for beta power (i.e., dominated by medial parietal areas). As a sensitivity analysis, and to ensure that the smaller PMBR and lower mean beta burst rates (during task blocks) observed for patients than controls were not due to poorer detection of beta bursts in patients due to use of a sub-optimal ICA component for the patient group, we repeated key analyses using a group ICA component derived from patient data only (see *Beta-burst analyses based on patient EEG only*).

Figure S1

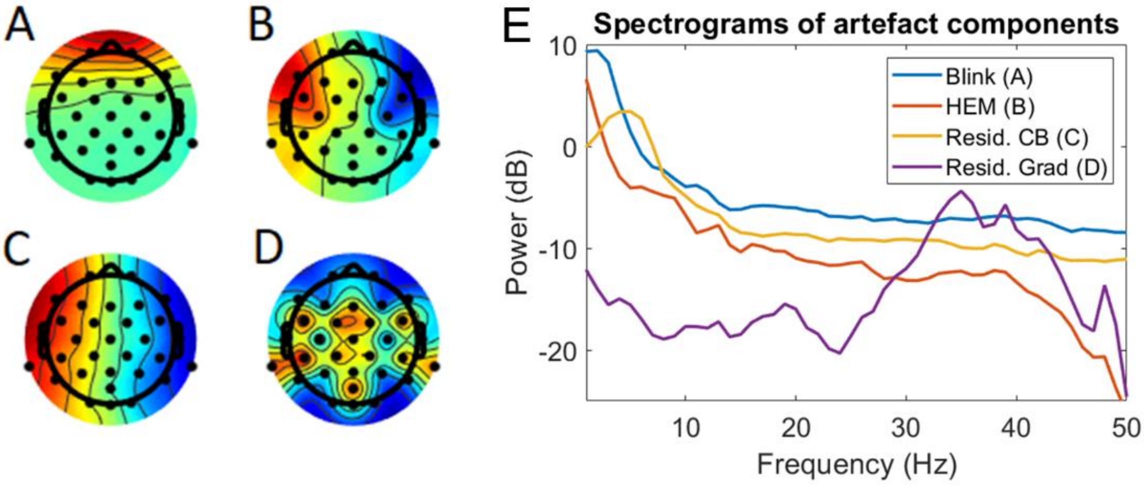

Example topographies of eye blink (A), horizontal eye movement – "HEM" (B), cardio-ballistic artefact – "CB" (C), and gradient artefact (D) ICA components, selected from those of a single participant, and their frequency spectrograms (E).

Since ICA was computed after gradient and cardio-ballistic artefact correction, the spectra in panel E are the spectra of the residue of these artefacts in the EEG data post-correction. Note, therefore, the already low power of these components (particularly in the beta band) prior to the removal of these components in pre-processing step 7 (see EEG acquisition and pre-processing).

fMRI acquisition and pre-processing

A 3T Philips Achieva MRI scanner, with an eight-channel SENSE head coil (SENSE factor 2 in anterior-posterior direction), was used to acquire gradient-echo echo-planar images. A dual-echo setup was used to enhance sensitivity, with echo times of TE_1/TE_2 20/48 ms, (flip angle 85°, 255 x 255 mm field of view, in-plane resolution 3 x 3 mm and slice thickness 4 mm). The repetition time (TR) was 2.5 seconds. 410 volumes, consisting of 32 contiguous axial slices, were acquired in each of the two N-back runs. In addition, an MPRAGE structural image was obtained for each participant for co-registration (1 mm isotropic resolution, TE 8.1 ms, TR 3.7 ms, SENSE factor 2, flip angle 8°, shot interval 3 seconds). Cardiac electrical activity was acquired at 500 Hz using an MR-compatible four-channel vector cardiogram (VCG) system (Philips, Netherlands) which also identified and stored the locations of R-wave peaks (used for cardio-ballistic artefact correction of the EEG recordings).

SPM 12 (13) was used for BOLD fMRI pre-processing and data analysis. The first five volumes collected in each run were discarded. Data were reoriented such that the anterior and posterior commissures lay on a horizontal plane, realigned to the first image in the time series, and slice-time corrected to the middle slice. The two echo times were then combined using a weighted sum of the measured time courses (14). Head movement and physiological noise were corrected using the RETROICOR toolbox (15). The mean BOLD image was then co-registered to the standard Montreal Neurological Institute (MNI) 152 brain (16) and the derived transform was applied to all the BOLD images. Spatial smoothing with a Gaussian kernel (8 mm FWHM) was performed. Volumes with significant artefact (scan-to-scan head motion of 0.5 mm or greater; outliers more than 3 SD from the global mean signal) were

removed and replaced using an interpolative method implemented in the ArtRepair toolbox (https://cibsr.stanford.edu/tools/human-brain-project/artrepair-software.html) (17).

First-level general linear models were constructed for each participant, incorporating both of their data runs. Design matrices included 0-back, 1-back, and 2-back sub-blocks modelled as rectangles convolved with the SPM canonical haemodynamic response function, as well as motor responses and beta-bursts modelled as impulses convolved with the same function. The first temporal derivatives of each of these time series were also included as predictors in the design matrices. The six realignment parameters were converted to 24 parameters as per Friston et al. (18) and included along with white matter, cerebrospinal fluid, and global signal regressors. Beta-bursts occurring during rest and during task periods were modelled separately, then equally weighted to form a first-level contrast of all beta-bursts versus baseline.

First-level images for the beta-event above baseline contrasts were entered into second-level, random effects, analyses. To estimate between-subject parameter values for voxels with missing data across participants, we used a multiple imputations procedure with five imputations, subject to the condition that data were present for at least 85% of participants for that voxel (19) (https://github.com/pmbriley/holeFiller). Second level analyses used a significance threshold of $p < 0.05$ with family-wise error rate correction for multiple comparisons, and with equal weighting given to the patient (schizophrenia and bipolar disorder groups considered together) and control groups. For identifying significant clusters for further analyses, a minimum cluster size of twenty was also applied. As a control analysis, we repeated the above with beta burst events replaced by fake events with the same density as beta bursts (averaged over each time period of twenty consecutive bursts). There were no

positive BOLD correlates of these fake events (even without a minimum cluster size); the negative BOLD correlates consisted of only two, non-continuous, posteriorly located, voxels.

To establish whether the BOLD correlates of beta-bursts represented reinforcement or suppression of any effects of task (overall effect of task block; effects of motor responses), we conducted a second series of analyses using a more lenient voxel-level threshold (p<.001, uncorrected) with a minimum cluster significance threshold of p<.05, FDR corrected. Clusters were obtained for effects of task block; motor responses; beta-bursts produced during task; and beta-bursts produced during rest. We tabulated the coordinates of the peak voxel in each of the significant clusters (positive and negative) for each of these four effects, which yielded 36 peak voxels, 35 of which met a voxel-level threshold of p<.05, FDR corrected (Table S1).

Table S1: Peak voxel coordinates for significant clusters

Effect	Sign	Peak signif. voxel (MNI co-ordinates)	AAL atlas regions
Rest beta-bursts	Positive	-18, -60, -24	Cerebellum 6 L
		-48, -30, 9	Temporal Sup L
		57, 3, 12	Rolandic Oper R
		12, -6, 69	Supp Motor Area R
	Negative	-9, -96, 0	Occipital Mid L
		51, -51, 27	Angular R
Task beta-bursts	Positive	-48, -12, 30	Postcentral L
		57, -9, 21	Postcentral R
		27, -30, 60	Postcentral R
	Negative	-36, -39, 39	Parietal Inf L
		42, -39, 39	SupraMarginal R
Motor responses	Positive	-48, -21, 48	Postcentral L
		-12, -18, 3	Thalamus L
		18, -54, -30	Cerebellum 6 R
		42, 3, -6	Insula R
		60, -33, 30	SupraMarginal R
	Negative	-45, -75, -12	Occipital Inf L
		42, -69, -12	Occipital Inf R
Task block	Positive	30, -57, 42	Angular R
		-42, -63, -15	Fusiform L
		-30, 24, -3	Insula L
		-33, -51, 48	Parietal Inf L
		-3, 3, 57	Supp Motor Area L
		30, -63, -33	Cerebellum Crus1 R
		36, 39, 21	Frontal Mid R
		33, 24, -3	Insula R
		51, 3, 45	Precentral R
		15, -9, -3	Thalamus R
	Negative	0, 54, 18	Frontal Sup Medial L
		-36, -21, 12	Insula L
		-6, -54, 9	Precuneus L
		-48, -63, 21	Temporal Mid L
		-54, 6, -33	Temporal Mid L
		48, -60, 24	Angular R
		30, -93, -9	Occipital Inf R

Peak voxel coordinates (p<.001, uncorrected) for significant clusters (p<.05 FWE corrected) for each of the four effects of interest, with the sign of the regression coefficient shown, and with associated AAL atlas labels.

BOLD correlates of beta-bursts without motor response regressors

Whilst the occurrence of beta bursts will be correlated with the occurrence of motor responses (due to the PMBR phenomenon), bursts and responses are not co-linear, since bursts occur throughout a run, including during rest periods (as evidenced, for example, in Figure 3F). The results reported in this paper used fMRI design matrices that incorporated motor responses, to conservatively account for the confounding effects of BOLD activation related to motor responses on observed BOLD activation related to beta bursts. To examine the effects of including motor responses in the fMRI design matrices on our key results, we also re-ran our fMRI analyses without a motor response regressor. Figure S2: A, B shows the resulting positive BOLD correlates of beta bursts, which can be directly compared with Figure 4: B, C. Differences in these activation maps are minimal. Figure S2C then shows the amount of BOLD activation associated with beta-bursts within each of the three clusters of activity identified in the main text, for controls and patients separately. This can be directly compared with Figure 6A. Again, differences are small. Importantly, patients continue to show significantly greater BOLD activation to beta bursts than controls [$F(1, 76) = 10.980$, $p = 0.001$, partial $\eta^2 = 0.13$].

Figure S2

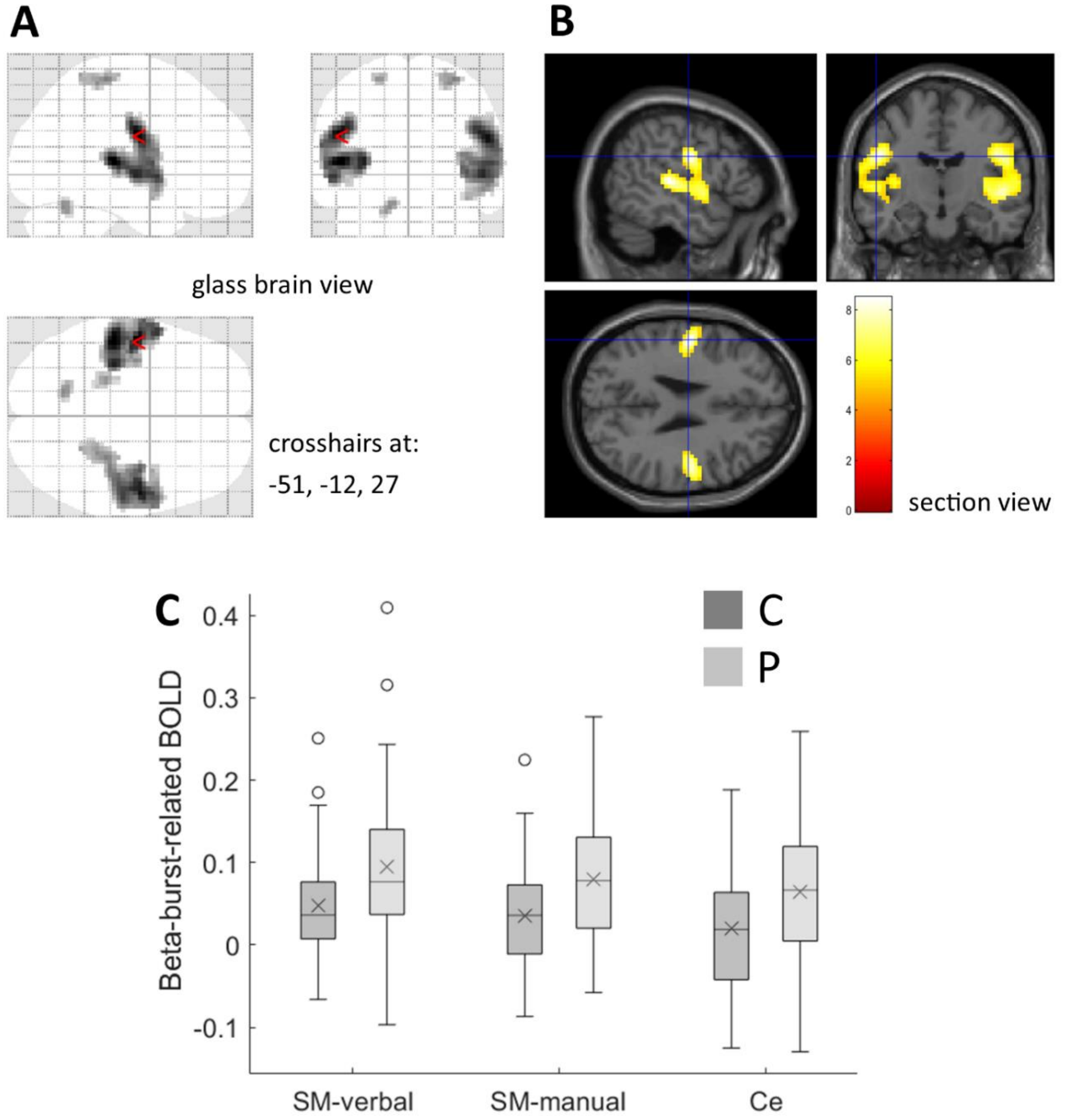

Positive BOLD correlates of beta bursts (A-B), and differences in the size of beta-burst-related BOLD activation between controls and patients (C), using an fMRI design matrix that did not include motor responses.

Panels A-B: As in Figure 4, clusters (k>=20) showing significant increases in BOLD activity associated with beta-bursts (cluster threshold p < 0.05, FWE corrected; voxel threshold p < 0.05, FDR corrected), plotted on an axial 5mm slice view overlaid on the SPM single subject brain (A) or on the SPM "glass brain" in three orthogonal planes (B). Panel C: As in Figure 6, boxplots showing distribution of BOLD activation associated with beta-bursts within SM-verbal (sensorimotor-verbal), SM-manual (sensorimotor-manual), and Ce (left cerebellar) ROIs. Upper and lower bounds of box denote Inter-quartile range (IQR); horizontal line denotes median; X denotes mean; whiskers denote range if within 1.5 x IQR; datapoints outside this range are shown as circles. Controls (C) versus patients (P).

BOLD correlates of beta-bursts relative to task and motor responses

Regions generally more active during task than rest included regions of two well-described task-positive networks (20) – the fronto-parietal executive control network also known as the Dorsal Attention Network (DAN) (21), and the Salience Network (SN) (22). Those showing reduced activity during the task included regions of the Default Mode Network (DMN) (Figure S3A, top row). Motor responses were associated with increased BOLD in contralateral motor cortex and ipsilateral cerebellum, and decreased BOLD in visual cortex (Figure S3A, bottom row).

As noted in the main text, regions positively correlated with beta-bursts tended to overlap with regions that showed significantly less activity during task than rest. That is, beta-bursts, whether produced during task or rest, tended to increase activity in regions otherwise suppressed during the N-back task (Figure S3B). In contrast, task beta-bursts suppressed regions of the task-positive posterior DAN (bilateral superior parietal sulcus) (Figure 5B, top row), while rest beta-bursts suppressed regions of the task-negative DMN including angular gyrus, middle temporal gyrus, and medial frontal gyrus (Figure 5B, bottom row). In addition, rest beta-bursts suppressed visual regions also suppressed by motor responses made during the task (Figure S3C).

Figure S3

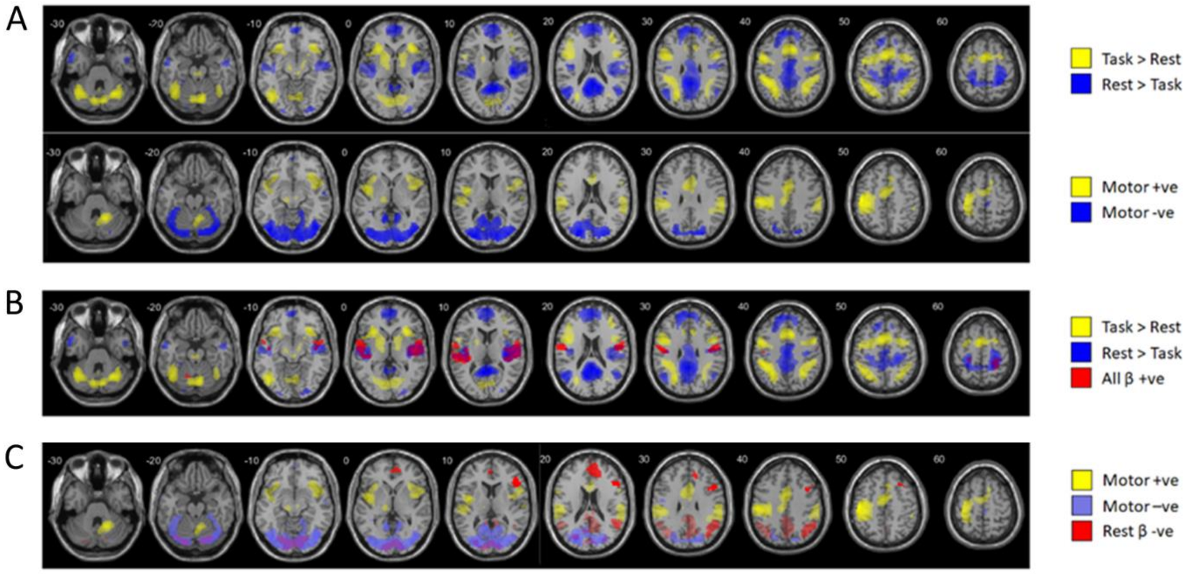

BOLD correlates of beta bursts compared to BOLD correlates of task.

All clusters shown are of voxels significant at p<.001, uncorrected, in clusters significant at p<.05, FWE corrected. Panel A: Positive and negative correlates of task block (top row) and motor responses (bottom row). Panel B: Positive correlates of all beta-bursts overlaid on positive and negative correlates of task block; beta-bursts, whether produced in task or rest, activated regions (shown in blue) that were otherwise suppressed during the task. Panel C: Negative correlates of rest beta-bursts overlaid on positive and negative correlates of motor response; beta-bursts produced during rest suppressed the same visual regions as those suppressed by a motor response during the task.

To further characterise beta-burst effects on BOLD signals relative to the BOLD contrasts associated with task activity, we obtained the regression coefficients for each of the four effects of interest (task-block; motor responses; task beta-bursts; rest beta-bursts) at the 35 voxels representing all the cluster maxima (Table S1). We then examined the regression coefficients for each effect at not only the cluster peaks for that effect, but for cluster peaks for each of the other effects. Specifically, we plotted the coefficients for beta-burst effects against coefficients for task effects, in order to reveal any overall tendency for beta-bursts to reinforce, or to counteract, task effects.

Regression coefficients for beta-bursts produced during task were strongly and negatively correlated with coefficients for task-blocks (Figure S4A), $r(33)=-.686$, $p<.001$, reflecting the pattern seen in Figure 5B (upper row), of task beta-bursts tending to counteract the overall effects of the task itself, increasing the BOLD signal in voxels showing overall suppression, and reducing it in regions showing overall activation. There was no significant correlation between rest beta-burst coefficients and task-block coefficients, $r(33)=.111$, $p=.509$, reflecting the pattern shown in Figure 5B (bottom row), namely that while beta-bursts produced during rest activated the same task-negative regions as those activated by task beta-bursts, they also tended to deactivate other task negative regions, including regions of the DMN.

In contrast, rest beta-burst coefficients were positively correlated with motor response coefficients (Figure S4B), $r(33)=.360$, $p=.033$. This suggests that at rest, beta-bursts elicit a similar pattern of activation and deactivation to that elicited by motor responses during the task. We saw no significant correlation between task beta-burst coefficients and those for motor responses, $r(33)=-.065$, $p=.711$, possibly because of the substantial temporal overlap between task beta-bursts and motor responses during task-blocks.

Figure S4

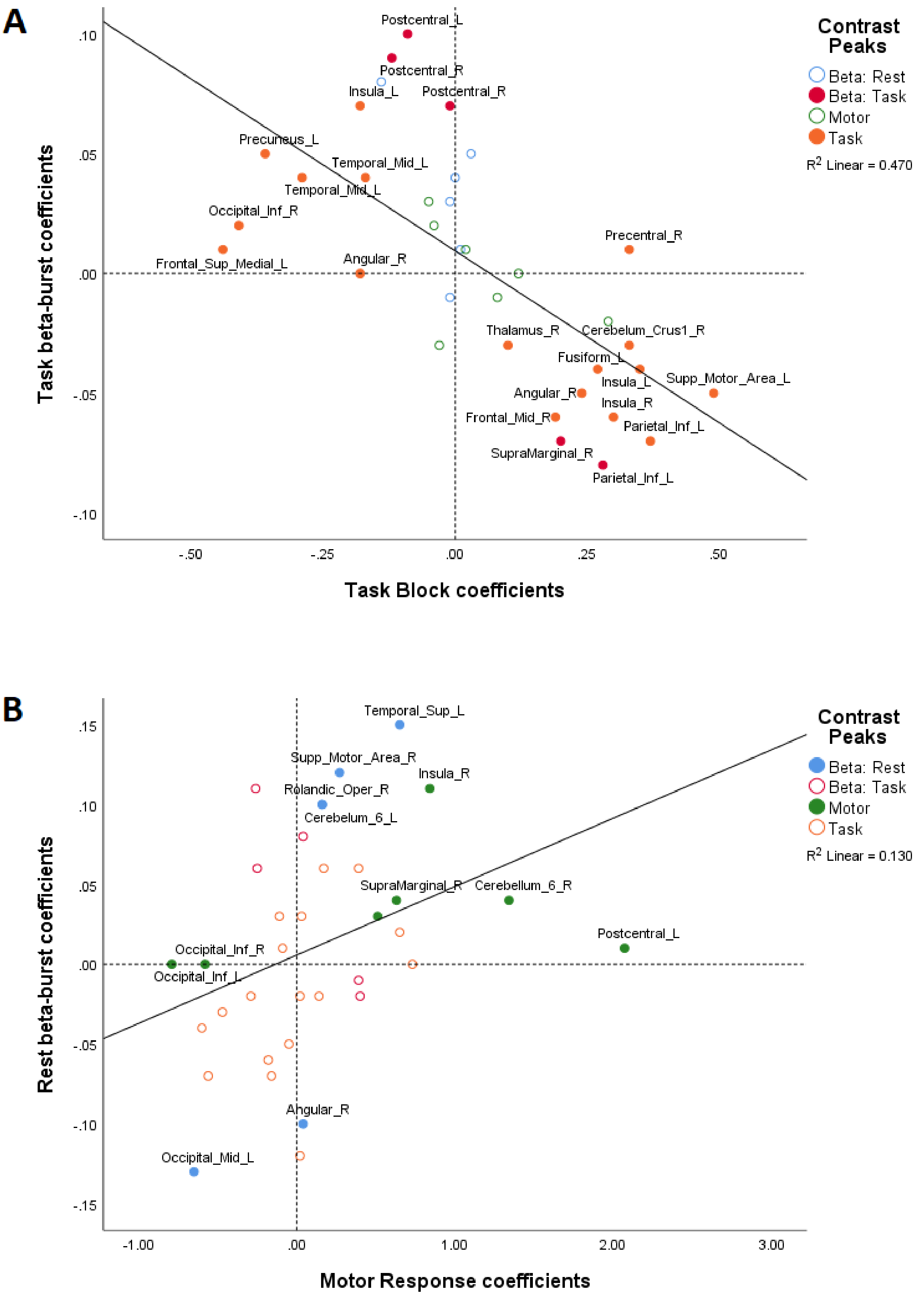
A
Contrast Peaks
Beta: Rest
Beta: Task
Motor
Task
R2 Linear = 0.470
Task beta-burst coefficients
Task Block coefficients
.10
.05
.00
-.05
-.10
-.50
-.25
.00
.25
.50
Postcentral_L
Postcentral_R
Insula_L
Postcentral_R
Precuneus_L
Temporal_Mid_L
Temporal_Mid_L
Occipital_Inf_R
Frontal_Sup_Medial_L
Angular_R
Precentral_R
Thalamus_R
Cerebelum_Crus1_R
Fusiform_L
Insula_L
Supp_Motor_Area_L
Angular_R
Insula_R
Frontal_Mid_R
Parietal_Inf_L
SupraMarginal_R
Parietal_Inf_L
B
Contrast Peaks
Beta: Rest
Beta: Task
Motor
Task
R2 Linear = 0.130
Rest beta-burst coefficients
Motor Response coefficients
.15
.10
.05
.00
-.05
-.10
-.15
-1.00
.00
1.00
2.00
3.00
Temporal_Sup_L
Supp_Motor_Area_R
Insula_R
Rolandic_Oper_R
Cerebelum_6_L
SupraMarginal_R
Cerebellum_6_R
Postcentral_L
Occipital_Inf_R
Occipital_Inf_L
Angular_R
Occipital_Mid_L

Scatterplots showing the relationships between regression coefficients for each of four effects of interest (task-beta; rest-beta, task block, motor response) at each of 35 voxels representing a cluster peak for one of the four effects of interest.

Panel A: Task beta-burst coefficients plotted against task block coefficients. Filled circles represent peaks in task beta-burst clusters (red) and task block clusters (orange) and the AAL region in which each voxel occurs is given. Open circles represent rest beta-burst and motor response cluster peaks (AAL region names not shown). Task beta-burst coefficients were strongly negatively correlated with task-block coefficients across this set of voxels, indicating that beta-bursts produced during task tended to counteract, rather than reinforce, the effect of task block across the full set of voxels. Panel B: Rest beta-burst coefficients plotted against motor response coefficients. Filled circles represent peaks in rest beta-burst clusters (blue) and motor response clusters (green) and the AAL region in which each voxel occurs is given. Open circles represent task beta-burst and task block cluster peaks. Rest beta burst coefficients were strongly positive correlated with coefficients for motor responses, indicating that beta bursts produced during rest blocks elicited a similar pattern of activation and deactivation to that elicited by motor responses during the task.

Beta-burst analyses based on patient EEG only

In the main analyses, we used a (group) independent component analysis (ICA) of cleaned EEG data from all participants to find a representative source of beta-frequency brain activity. This was applied to the data from each participant to identify beta bursts, ensuring comparability across participants (see *EEG acquisition and pre-processing*). It is possible, however, that the smaller PMBR, and lower mean beta burst rates during task, that we observed for patients than controls (Figure 3) may reflect poorer detection of beta bursts in patients due to selection of a sub-optimal ICA component for this group. As a sensitivity analysis, we repeated the group ICA solely on the patient data. A similar ICA component to that previously chosen was seen (Figure S5A) – the weights of this component were applied to the cleaned data from each participant, and beta bursts were identified with the same parameters as used previously.

Beta burst rate, calculated in sliding time windows, for patients and controls using this new group component is shown in Figure S5B (compare Figure 3A), and PMBR for controls and patients, and for patients with bipolar disorder and patients with schizophrenia, is shown in Figure S5C (compare Figure 3D). Mean burst rates, calculated during rest, 0-back, 1-back, and 2-back blocks, is shown in Figure S5D (compare Figure 3F). As in the main analyses, PMBR was significantly attenuated in patients relative to controls [$F(1, 76) = 6.266$, $p = 0.014$, partial $\eta^2 = 0.08$]. Likewise, mean beta-burst rate decreased monotonically with increasing working memory load, [$F(3,228) = 30.852$, $p < 0.001$, partial $\eta^2 = 0.29$], with this reduction with load being greater in patients than controls [$F(3,228) = 3.767$, $p = 0.011$, partial $\eta^2 = 0.05$]. The positive BOLD correlates of beta bursts identified using this component were similar to those seen in the main analyses (data not shown). Figure S5E shows the amount of BOLD activation associated with beta-bursts within each of the three clusters of activity identified in the main text, for controls and patients separately. This can be directly compared with Figure 6A. Results were similar to those from the main analyses, and patients continued to show significantly greater BOLD activation to beta bursts than controls [$F(1, 76) = 7.221$, $p = 0.009$, partial $\eta^2 = 0.09$].

Figure S5

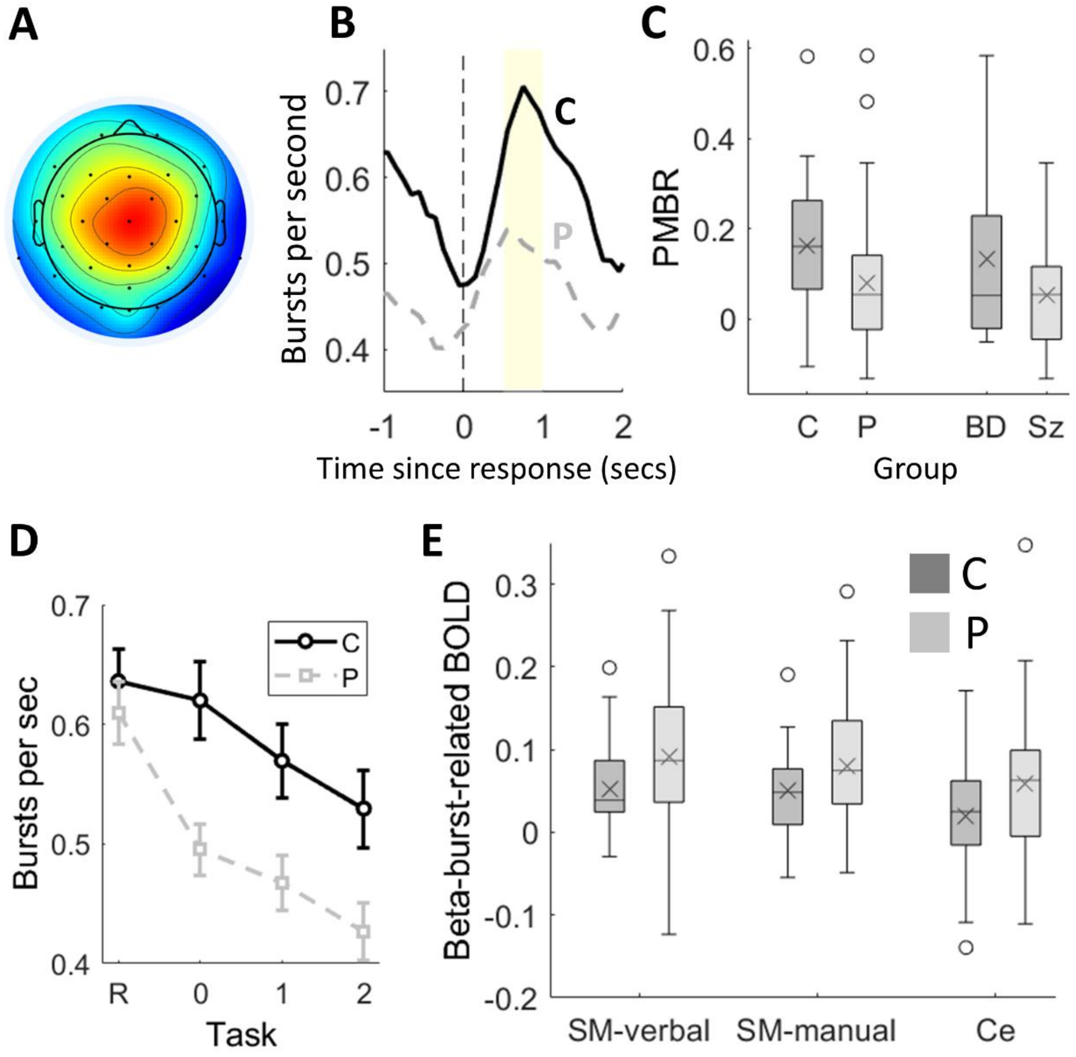

Impact on estimates of beta burst rate and beta-burst-related BOLD activity of using a group ICA component derived from patient data only.

Panel A: Topography of the chosen ICA component, selected from a group ICA of cleaned EEG data from patients only. Panel B: Mean beta-bursts per second calculated in 500 ms sliding time windows for controls ("C", solid black line) and patients ("P", dashed grey line), time locked to motor responses (the shaded region indicates the PMBR window). Panel C: Boxplots showing the distributions of PMBR for controls versus patients and for patients with bipolar disorder (BD) versus patients with schizophrenia/schizoaffective disorder (Sz), collapsed across task conditions. Upper and lower bounds of box denote Inter-quartile range (IQR); horizontal line denotes median; X denotes mean; whiskers denote range if within 1.5 x IQR; datapoints outside this range are shown as circles. *(D) Mean beta-burst rate for controls and patients for rest ("R"), 0-back, 1-back and 2-back conditions. Error bars show ± 1 S.E.M. (E) Boxplots showing distribution of BOLD activation associated with beta bursts within SM-verbal (sensorimotor-verbal), SM-manual (sensorimotor-manual) and Ce (left cerebellar) ROIs.*

PERSISTENCE OF SIGNS AND SYMPTOMS IN PSYCHOTIC ILLNESS SCORING FORM

A	EPISODE NUMBER									
B	DURATION (with month & year)									
C	HOSPITALISATION (Y/N)									
D	MEDICATIONS<br>Antipsychotics (Max dose)<br>Mood stabilisers (Name)<br>Antidepressants (Y/N)									
E	CLUSTERS									Persistence score
E 1	Anxiety/Depression									
E 2	Psychomotor poverty<br>Underactivity, Flat affect, Poverty of speech and Anhedonia									
E 3	Psychomotor excitation<br>Elation, Overactivity, Pressure of speech, Insomnia, Hostility and Peculiar behaviour									
E 4	Disorganisation<br>Inappropriate affect, Formal thought disorder, Impaired attention, Disorientation									
E 5	Reality Distortion<br>Delusions, Hallucinations, Somatisation									

Notes for scoring:

A Episode number: An episode is a period when mental symptoms are present for a period of at least one week and global mental state (as defined by GAF criteria) is appreciably worse than usual, e.g. more than 10 points drop in GAF score; episodes that are less than 6 months apart do not count as separate episodes.

B Duration: Estimate of month & year of onset and remission of each episode.

D Medications: Indicate antipsychotic medications used with maximum dose achieved in chlorpromazine equivalents.

E Clusters

0	Indicates definite absence of the symptoms judged from 1 or more sources within a case record.
1	Refers to questionable presence of the symptoms judged from 1 or more sources within a case record.
2	Refers to definite presence of the symptoms judged from 1 or more sources within a case record.
9	Is applied ONLY IF NO information could be gathered at any time period regarding this cluster.

Anxiety/depression Symptoms of anxiety to be scored here only if not explained by reality distortion experiences or normal physiological response to obvious stressors

Psychomotor poverty Anhedonia contributes to psychomotor poverty. Any of the features of psychomotor poverty can occur within a depressive episode; the definition of depression is lowered mood, pessimism and low self-esteem.

Persistence score

1. Clinically significant mental symptoms recorded at only one assessment*
2. Symptoms recorded at multiple assessments* but evidence indicates clear remission between episodes
3. There is evidence indicating continuous presence of at least some symptoms during inter-assessment periods in the early stages of illness; however, in the later stages of the illness there is clear remission between the episodes
4. Complete remission during the early stages, however during the later stages there is evidence indicating continuous presence of at least some symptoms during inter-episode periods
5. There is evidence indicating continuous presence of at least some symptoms during most inter-episode periods ever since the first episode
6. Chronic symptoms with evidence that the majority of symptoms have been continually present ever since the first episode
8. Not enough information available in the notes to score

* The most informative assessment within any 6-month period will be employed (e.g., admission clerking, outpatient clinic, Mental Health Act assessment or care program review)

SUPPLEMENTAL REFERENCES

1. Ammons RB, Ammons CH (1962): The Quick Test (QT): Provisional manual. *Psychol Rep* 11: 111–161.

2. American Psychiatric Association (1994): *Diagnostic and Statistical Manual of Mental Disorders*, 4th ed. Washington DC: American Psychiatric Association.

3. Liddle PF, Ngan ETC, Duffield G, Kho K, Warren AJ (2002): Signs and Symptoms of Psychotic Illness (SSPI): a rating scale. *Br J Psychiatry* 180: 45–50.

4. The MathWorks, Inc. (2019): *MatLab*, version 2019b.

5. Delorme A, Makeig S (2004): EEGLAB: an open source toolbox for analysis of single-trial EEG dynamics including independent component analysis. *J Neurosci Methods* 134: 9–21.

6. Iannetti GD, Niazy RK, Wise RG, Jezzard P, Brooks JCW, Zambreanu L, *et al.* (2005): Simultaneous recording of laser-evoked brain potentials and continuous, high-field functional magnetic resonance imaging in humans. *NeuroImage* 28: 708–719.

7. Niazy RK, Beckmann CF, Iannetti GD, Brady JM, Smith SM (2005): Removal of FMRI environment artifacts from EEG data using optimal basis sets. *NeuroImage* 28: 720–737.

8. Bell AJ, Sejnowski TJ (1995): An Information-Maximization Approach to Blind Separation and Blind Deconvolution. *Neural Comput* 7: 1129–1159.

9. Lee T-W, Girolami M, Sejnowski TJ (1999): Independent Component Analysis Using an Extended Infomax Algorithm for Mixed Subgaussian and Supergaussian Sources. *Neural Comput* 11: 417–441.

10. Makeig S, Bell AJ, Jung T-P, Sejnowski TJ (1996): Independent component analysis of electroencephalographic data. In: Touretsky D, Mozer M, Hasselmo M, editors. *Advances in Neural Information Processing Systems*. Cambridge, MA, US: MIT Press, pp 145–151.

11. Mayeli A, Zotev V, Refai H, Bodurka J (2016): Real-time EEG artifact correction during fMRI using ICA. *J Neurosci Methods* 274: 27–37.

12. Raimondo F, Kamienkowski JE, Sigman M, Fernandez Slezak D (2012): CUDAICA: GPU Optimization of Infomax-ICA EEG Analysis. *Comput Intell Neurosci* 2012. https://doi.org/10.1155/2012/206972

13. https://www.fil.ion (2020): *SPM12*.

14. Gowland PA, Bowtell R (2007): Theoretical optimization of multi-echo fMRI data acquisition. *Phys Med Biol* 52: 1801–1813.

15. Glover GH, Li T-Q, Ress D (2000): Image-based method for retrospective correction of physiological motion effects in fMRI: RETROICOR. *Magn Reson Med* 44: 162–167.

16. Mazziotta J, Toga A, Evans A, Fox P, Lancaster J, Zilles K, *et al.* (2001): A probabilistic atlas and reference system for the human brain: International Consortium for Brain Mapping (ICBM). *Philos Trans R Soc Lond Ser B* 356: 1293–1322.

17. Mazaika PK, Hoeft F, Glover GH, Reiss AL (2009): Methods and software for fMRI analysis of clinical subjects. *NeuroImage* 47: S58.

18. Friston KJ, Williams S, Howard R, Frackowiak RS, Turner R (1996): Movement-related effects in fMRI time-series. *Magn Reson Med* 35: 346–355.

19. Liddle EB, Bates AT, Das D, White TP, Groom MJ, Jansen M, *et al.* (2013): Inefficient cerebral recruitment as a vulnerability marker for schizophrenia. *Psychol Med* 43: 169–182.

20. Fox MD, Snyder AZ, Vincent JL, Corbetta M, Van Essen DC, Raichle ME (2005): The human brain is intrinsically organized into dynamic, anticorrelated functional networks. *Proc Natl Acad Sci U S A* 102: 9673–9678.

21. Dosenbach NUF, Fair DA, Miezin FM, Cohen AL, Wenger KK, Dosenbach RAT, *et al.* (2007): Distinct brain networks for adaptive and stable task control in humans. *Proc Natl Acad Sci* 104: 11073–11078.

22. Seeley WW, Menon V, Schatzberg AF, Keller J, Glover GH, Kenna H, *et al.* (2007): Dissociable Intrinsic Connectivity Networks for Salience Processing and Executive Control. *J Neurosci* 27: 2349–2356.
